# Dynamics of Haemostatic and Inflammatory Biomarkers in Patients with Combat-Related Injuries to Major Joints Before and After Surgical Treatment

**DOI:** 10.3390/jcm15010322

**Published:** 2026-01-01

**Authors:** Stanislav Bondarenko, Alfonso Alías Petralanda, Yuriy Prudnikov, Beniamin Oskar Grabarek, Dariusz Boroń, Piotr Ossowski, Volodymyr Filipenko, Frida Leontjeva, Vladislav Tuljakov, Fedir Klymovytskyy

**Affiliations:** 1 Sytenko Institute of Spine and Joint Pathology, 80 Hryhoriia Skovorody Str., 61024 Kharkiv, Ukraine; bondarenke@gmail.com (S.B.); filippenko1957@gmail.com (V.F.); frida@farfora.com (F.L.); osterixy@ukr.net (V.T.); 2 Hospital Clinic Barcelona, C/Villarroel, 170, 08036 Barcelona, Spain; alias@clinic.cat; 3 Collegium Medicum, WSB University, 1C Zygmunt Cieplak Str., 41-300 Dabrowa Gornicza, Poland; bgrabarek7@gmail.com (B.O.G.); dariusz@boron.pl (D.B.); drpiotrossowski@gmail.com (P.O.); 4 Research Institute of Traumatology and Orthopedics, Donetsk National Medical University, 36-a Hasieva Str., 84401 Lyman, Ukraine; klimovitskiyfedor@gmail.com

**Keywords:** combat injury, haemostasis, fibrinolysis, inflammation, hypercoagulability, biomarkers, orthopaedic trauma, joint surgery, thromboinflammation

## Abstract

**Background/Objectives**: Combat trauma involving large joints is associated with a high risk of thromboinflammatory complications. Early identification of laboratory markers for hypercoagulability is essential to optimise perioperative management. This study aimed to evaluate the dynamics of inflammation and haemostasis indicators in patients with combat-related joint trauma and to identify the most informative markers for preoperative risk assessment. **Methods**: A total of 29 patients with combat injuries to the hip, knee, elbow, or ankle joints were examined. Blood samples were taken 1–3 days prior to surgery and again on the first postoperative day. Parameters of coagulation (e.g., PT, INR, fibrinogen, D-dimer, soluble fibrin complexes, antithrombin III), fibrinolysis, and inflammation (e.g., CRP, haptoglobin, sialic acid, ESR, LSI, LII) were analysed and compared to those of 30 healthy controls. Statistical analysis included Student’s *t*-test and Pearson’s correlation. **Results**: At baseline, patients demonstrated significant increases in inflammatory markers (CRP 64.2 ± 7.3 mg/L, ↑738.9%; haptoglobin 3.25 ± 0.4 g/L, ↑164.3%; ESR 46.8 ± 5.2 mm/h, ↑313.8%) and procoagulant activity (D-dimer 1.42 ± 0.18 µg/mL, ↑136.6%; fibrinogen 6.12 ± 0.51 g/L, ↑102.4%; soluble fibrin complexes 38.7 ± 4.9 mg/L, ↑597.3%), together with a reduction in antithrombin III activity (63.5 ± 6.2%, ↓39.5%) and prolonged fibrinolysis time (increase by 197%). Postoperatively, these abnormalities intensified, indicating a sustained thromboinflammatory response. Strong correlations were found between inflammatory and haemostatic markers. **Conclusions**: Combat trauma of large joints is associated with preoperative thromboinflammatory dysregulation, which is exacerbated by surgery. Monitoring specific biochemical and haematological markers—such as CRP, fibrinogen, D-dimer, and soluble fibrin complexes—may support preoperative risk assessment and postoperative monitoring strategies for hypercoagulable states in this high-risk group. These findings lay the groundwork for future prospective studies aimed at developing stratified therapeutic protocols and predictive models for thromboinflammatory complications in orthopaedic trauma care.

## 1. Introduction

Inflammation and coagulation are two fundamental protective systems that are intricately interconnected, serving to limit tissue damage and support the restoration of homeostasis following injury [1]. The bidirectional interplay between these systems is of particular importance in trauma patients, where dysregulation can contribute to the development of multiple organ dysfunction syndrome (MODS) [2].

Recent studies have highlighted a strong association between proinflammatory mediators—particularly interleukin-6 (IL-6)—and coagulation pathways, indicating a shared pathophysiological axis that contributes to thromboinflammatory responses in severe conditions such as COVID-19 and systemic inflammation [3,4]. Central to this interface is the vascular endothelium, which plays a key role in orchestrating both thrombin generation and fibrinolysis inhibition. Endothelial cells respond to inflammatory stimuli by expressing adhesion molecules, cytokines, and procoagulant factors such as tissue factor (TF), thus amplifying coagulation via the extrinsic (TF–Factor VIIa) pathway, rather than the intrinsic (contact activation) route [2].

Moreover, injury to the endothelium exposes subendothelial structures, including collagen and TF, which can trigger the coagulation cascade, promoting thrombus formation [5]. Activated platelets are also critical mediators at the crossroads of coagulation and inflammation. By expressing phosphatidylserine and P-selectin, they not only enhance thrombin generation but also release a host of cytokines and chemokines, thereby contributing to the inflammatory milieu and the formation of microvascular thrombi [6].

The fibrinolytic system, which serves to dissolve fibrin clots and restore vessel patency, is an essential counterbalance to coagulation. As reviewed by Hvas and Larsen [7], advances in the understanding and measurement of fibrinolytic markers have provided valuable insight into the dysregulation of this system during inflammation and trauma. The concept of “fibrinolysis shutdown”—where fibrinolytic activity is markedly suppressed—has been described in patients with severe trauma and is believed to contribute to poor outcomes due to persistent microvascular thrombosis and thromboinflammatory responses.

Natural anticoagulants such as antithrombin play a dual role in modulating coagulation and inflammation. Antithrombin inhibits thrombin and Factor Xa and attenuates inflammation by suppressing platelet activation, reducing leukocyte–endothelial adhesion, and dampening cytokine production [8]. However, in trauma, its levels may decrease due to degradation by neutrophil elastase, reduced hepatic synthesis, or consumption [9]. Additionally, trauma-induced activation of protein C can contribute to increased clotting, hyperfibrinolysis, and fibrinogen depletion [10].

Inflammatory mediators like IL-6 can further activate coagulation by upregulating TF expression on monocytes and endothelial cells [11], while coagulation factors such as thrombin can reciprocally modulate immune responses via protease-activated receptors, contributing to cytokine release and leukocyte recruitment. Fibrin itself can also serve as a scaffold for immune cell adhesion and complement activation, thereby amplifying inflammation [12].

This intricate crosstalk among coagulation, the immune system, and the complement cascade can lead to coagulopathy, platelet dysfunction, and endotheliopathy—hallmarks of trauma-induced coagulopathy that require targeted perioperative management [11]. Complement components like C3a and C5a, generated via classical, lectin, or alternative pathways, modulate leukocyte activity and promote thrombosis [13], while tissue factor and Factor VIIa may also propagate inflammation [14].

Simultaneous elevation of key biomarkers from both coagulation and inflammatory pathways can serve as prognostic indicators of adverse outcomes [15]. Elevated D-dimer levels, in particular, have been linked to conditions such as deep vein thrombosis, pulmonary embolism, and disseminated intravascular coagulation [16]. Risk of postoperative venous thromboembolism (VTE) is notably increased when D-dimer exceeds 3.4–5.3 μg/mL [17]. Additional markers such as thrombin–antithrombin complexes and prothrombin fragments F1+2 may provide further insights into hypercoagulability [18], while the presence of soluble blood fibrin (SBF) has been proposed as a more specific predictor than D-dimer alone [19].

Recent research has further dissected how different orthopaedic interventions influence the balance between inflammation and coagulation. In a large-scale comparative study, Touw et al. [20] analysed data from over 1900 patients to assess the effects of lower-leg trauma and knee arthroscopy on coagulation parameters. Their findings indicate that lower-leg injuries are associated with significant elevations in procoagulant factors—including Factor VIII, von Willebrand factor (VWF), fibrinogen, and D-dimer—alongside increased interleukin-6 (IL-6), suggesting an acute systemic prothrombotic and inflammatory state. In contrast, patients undergoing knee arthroscopy exhibited a reduction in these markers postoperatively, indicating that distinct pathophysiological mechanisms underlie coagulation activation in trauma versus minimally invasive surgical procedures.

In the context of major joint surgeries, such as total hip and knee arthroplasty, the management of perioperative bleeding and inflammation remains critical. Tranexamic acid (TXA), an antifibrinolytic agent, is widely used to reduce intraoperative blood loss. Chen et al. [21] demonstrated in a randomized controlled trial that a three-dose regimen of TXA administered during total hip arthroplasty (THA) significantly attenuated postoperative inflammatory responses, minimized fibrinolysis—as evidenced by decreased fibrin degradation products (FDP)—and improved nutritional parameters such as albumin levels. This suggests that TXA not only modulates haemostasis but may also contribute to immune and metabolic stability in the postoperative period.

However, evidence regarding the inflammatory impact of TXA is not entirely consistent. In a pilot study on patients undergoing total knee arthroplasty (TKA), Grant et al. [22] observed that administration of TXA was paradoxically associated with a marked increase in systemic inflammatory mediators, including MCP-1, TNF-α, IL-1β, and IL-6, especially during the immediate postoperative period. Although TXA prolonged clotting times (EXTEM CT) and increased FIBTEM maximum lysis (ML)—a finding at odds with its expected antifibrinolytic profile—no significant changes in fibrinogen or postoperative haemoglobin levels were observed. These findings underscore the complexity of the coagulation–inflammation interface in orthopaedic surgery and suggest that the effects of TXA may be context-dependent, potentially differing between THA and TKA, or based on individual patient profiles.

Craven et al. [23] demonstrated that even before surgery, patients with chronic joint diseases exhibit elevated levels of procoagulant markers such as activated Factor VII and prothrombin fragment 1 + 2, likely due to the chronic inflammatory state. This preexisting prothrombotic condition, if unmitigated by natural anticoagulants such as TFPI, may predispose these patients to postoperative thromboembolic complications.

The extent of surgical trauma also modulates this balance. Pape et al. [24] showed that femoral nailing and total hip arthroplasty elicit comparable increases in IL-6 and prothrombin fragments, but the most pronounced responses occurred in multiply traumatized patients. Kristiansson et al. [25] distinguished between local and systemic cytokine release following hip arthroplasty, finding that local cytokine concentrations in wound drainage were significantly higher, suggesting a predominant local immune response despite systemic effects.

Similarly, Høgevold et al. [26] reported upregulation of leukocyte adhesion molecules and increases in IL-6, thrombin–antithrombin complexes (TAT), and C-reactive protein (CRP), underscoring the systemic activation of coagulation and inflammation following hip replacement. Their findings suggest that distinct cellular and molecular pathways independently regulate immune cell adhesion and acute-phase protein synthesis.

The integration of advanced technologies such as artificial intelligence (AI) into orthopaedic diagnostics offers additional tools to enhance the identification of patients at increased risk of complications. A recent review by Prudnikov et al. [27] underscores the expanding role of AI in analysing imaging and clinical data to improve diagnostic precision and guide treatment strategies in musculoskeletal disorders, including those with thromboinflammatory components.

Nevertheless, existing studies often overlook the unique pathophysiological context of combat-related joint injuries, where both inflammatory and haemostatic responses may be amplified. Such trauma is frequently characterised by polytrauma burden, delayed medical evacuation, high contamination rates, repeated surgical interventions, and extensive soft-tissue destruction—all factors known to intensify systemic inflammation and coagulation dysregulation. These circumstances make combat-related injuries distinct from civilian orthopaedic trauma and may modify the course of thromboinflammatory responses. Furthermore, the perioperative dynamics of key biomarkers in such patients remain insufficiently characterised, limiting the ability to stratify risk and individualise care.

In this context, the present study aims to address these knowledge gaps by characterising perioperative changes in inflammation and haemostasis markers in patients with combat-related joint trauma, using a pre-/postoperative design. The primary endpoint was defined as the perioperative change in coagulation and inflammatory biomarkers, with secondary analysis focusing on correlations between inflammatory and haemostatic parameters. “Most informative markers” were defined as those demonstrating the largest relative changes from baseline and the strongest inter-marker correlations, reflecting both biological and potential clinical relevance.

The purpose of this study is to assess changes in markers of haemostasis and inflammation in patients with combat-related large joint injuries, before and after surgical intervention, in order to identify the most informative parameters for preoperative assessment of hypercoagulable risk.

The structure of the present article reflects a systematic approach to investigating the biochemical underpinnings of thromboinflammatory risk in patients with combat-related joint trauma. Section 2 outlines the selection criteria for participants, the timing of perioperative blood sampling, and the analytical techniques employed to evaluate both coagulation and inflammatory markers. Section 3 presents significant perioperative alterations in fibrinogen levels, D-dimer, soluble fibrin complexes, and inflammatory indicators such as C-reactive protein and haptoglobin, alongside statistically significant correlations between inflammatory processes and hypercoagulability. Section 4 provides an in-depth interpretation of the observed haemostatic and inflammatory dynamics, contextualising the findings within the broader scientific literature. Finally, Section 5 identifies a set of laboratory markers most informative for the preoperative assessment of hypercoagulable risk and advocates for comprehensive monitoring protocols tailored to this high-risk patient cohort.

## 2. Materials and Methods

The study was conducted at the Clinic of the Sytenko Institute of Spine and Joint Pathology, National Academy of Medical Sciences of Ukraine (Accreditation Certificate, Higher Category, valid from 19 April 2021 to 18 April 2024, No. 015211, Series M3), in collaboration with the Department of Laboratory Diagnostics and Immunology of the Institute (Certificate of Compliance with DSTU ISO 10012:2005, valid from 14 March 2023 to 14 March 2026, No. 01-0017/2023).

This work is based on the biochemical analysis of data from 29 patients with combat-related injuries to the hip, knee, elbow, or ankle joints, treated at the Department of Joint Pathology of the Sytenko Institute. The study was carried out in accordance with national legislation regarding the inclusion of patients in clinical research. The study protocol was reviewed and approved by the Institute’s Bioethics Committee (Protocol No. 224 dated 13 June 2023). Informed consent was obtained from all participants using the form recommended by the Ministry of Health of Ukraine.

The inclusion criteria were patients with combat-related traumatic injuries of large joints who required surgical treatment. Exclusion criteria included refusal to participate in the study.

Venous blood samples were collected from the cubital vein into vacuum tubes containing sodium citrate for coagulation studies, K2-EDTA for complete blood counts, and plain tubes for serum preparation. Sampling was performed 1–3 days before surgery (preoperative) and on the first postoperative day, ensuring paired measurements in the same patients. For plasma analysis, blood was centrifuged at 3000 rpm for 15 min. Serum was obtained by clotting, followed by centrifugation at 1500 rpm for 30 min. Laboratory parameters measured included prothrombin time (PT), international normalised ratio (INR), soluble fibrin complexes (SFCs), D-dimer (DD), fibrinogen (FG), activated partial thromboplastin time (aPTT), and fibrinolytic activity (FA), using commercial reagent kits from Granum (Ukraine), in accordance with the manufacturer’s instructions.

Antithrombin III (AT-III) activity in plasma was assessed based on the residual thrombin activity following its interaction with antithrombin in defibrinated plasma, using Granum (Ukraine) reagents in accordance with the manufacturer’s instructions. Coagulometric parameters were measured using the automated coagulometric analyser Thrombolyzer Compact X (Behnk Elektronik GmbH & Co. KG, Norderstedt, Germany).

Serum concentrations of sialic acids (SAs) were determined using the Hess method; seromucoids (SGs) were measured by the turbidimetric method of Huergo; and haptoglobin (HG) levels were assessed via the rivanol precipitation method [28]. C-reactive protein (CRP) was semi-quantitatively determined using a latex agglutination test. All biochemical assays were performed using the semi-automated biochemical analyser GBG STAT FAX 1904 Plus (Awareness Technology, Inc., Palm City, FL, USA).

As part of the general haematological analysis, total leukocyte counts were measured using an automated haematology analyser SWELAB ALFA (Boule Diagnostics AB, Spånga, Stockholm, Sweden). Differential leukocyte counts (leukogram) were visually evaluated using a Motic BA 300 (Motic Incorporation Ltd., Hong Kong, China) microscope with Romanowsky–Giemsa staining. The erythrocyte sedimentation rate (ESR) was determined by the Westergren method [28]. In addition, the following integrated haematological indices were calculated [29]:

- Leukocyte shift index (LSI) = (myelocytes + metamyelocytes + band neutrophils + segmented neutrophils + eosinophils + basophils)/lymphocytes;
- Leukocyte index of intoxication (LII) = (myelocytes + metamyelocytes + band neutrophils + segmented neutrophils + plasma cells)/(lymphocytes + monocytes + eosinophils + basophils).

A control group consisting of 30 clinically healthy volunteers was included to provide a physiological reference baseline. All laboratory parameters in the control group and in patients with combat-related large joint injuries were assessed using identical analytical protocols, reagents, equipment, and pre-analytical conditions. This design enabled direct and methodologically consistent comparisons between the control cohort and the target patient group, allowing for an objective evaluation of trauma-associated alterations in haemostatic, inflammatory, and haematological markers.

Data analysis was performed using licensed MS Windows software package No. 439108-251. Normality of distribution was assessed using the Kolmogorov–Smirnov test. Results are presented as mean ± standard deviation (M ± m). Statistical comparisons within the patient group (pre- vs. postoperative values) were conducted using the paired Fisher–Student *t*-test. Comparisons between patients and healthy controls were performed using the unpaired *t*-test. A *p*-value < 0.05 was considered statistically significant. Correlations between parameters were evaluated using Pearson’s correlation coefficient (r) [30].

## 3. Results

Patients with combat-related joint trauma demonstrated a hypercoagulable state prior to surgical intervention. Specifically, prothrombin time (PT) was reduced by 19.51%, and the international normalised ratio (INR) by 14.92%, compared to the control group (Table 1). Fibrinogen (FG) levels were elevated, exceeding those of healthy individuals by 102.38%. These prothrombotic changes were accompanied by a 102.38% prolongation in fibrinolytic activity (FA) time, indicating a corresponding suppression of fibrinolysis.

Activation of the coagulation system was evidenced by a marked increase in the content of soluble fibrin complexes (SFCs) in the blood of patients, expressed in mg/L, which rose by 597.30% compared to the control group. Similarly, the concentration of another sensitive marker—D-dimer (DD)—was significantly elevated, increasing by 136.57%.

Conversely, antithrombin III (AT), a key physiological inhibitor of coagulation, was reduced by 39.52%, indicating consumption or degradation in the context of ongoing thrombin generation (Table 1).

Upon admission, patients also demonstrated significant elevations in serum markers of inflammation. Sialic acids (SAs) were increased by 64.40%, seroglycoids (SGs) by 73.68%, haptoglobin (HG) by 164.29%, and C-reactive protein (CRP) by 738.89% relative to the control group (Table 1).

Findings from general clinical blood analysis and leukocyte indices supported the presence of systemic inflammation. Erythrocyte sedimentation rate (ESR) exceeded control values by 313.83%, while the leukocyte shift index (LSI) and leukocyte index of intoxication (LII) were increased by 21.04% and 150.88%, respectively.

Following surgical intervention, further activation of haemostasis was observed. Fibrinogen (FG) levels rose an additional 44% compared to preoperative values, resulting in an overall increase of 190.48% versus the control group. Fibrinolytic activity (FA) was further impaired, as indicated by a prolongation of lysis time—20.88% longer than preoperative values and 259.00% above controls (Table 1).

Postoperatively, SFC levels (mg/L) continued to rise by 18.69% relative to preoperative values and were 727.63% higher than in the control group. D-dimer increased by 24.67% after surgery, reaching 194.93% above control values.

Inflammatory markers also remained elevated after surgery: SA by 81.15%, SG by 121.05%, HG by 205.41%, and CRP by 908.00% versus controls (Table 1).

ESR rose further, exceeding control values by 519.15%. Postoperative increases were also noted in LSI and LII, which were 47.33% and 190.70% higher than in the control group, respectively.

Surgical treatment resulted in an additional increase in inflammatory markers. Compared to preoperative values, SG increased by 27.27%, CRP by 20.11%, and ESR by 49.61%, while LSI rose by 21.72% (Table 1).

Comparative analysis revealed pronounced and statistically significant differences between patients with combat-related joint injuries and the control group across multiple haemostatic and inflammatory parameters. At admission, patients demonstrated marked deviations from control values, indicating a combined hypercoagulable and proinflammatory state. The magnitude of these differences, expressed as percentage changes relative to controls, is summarised in Table 1 and served as the basis for subsequent evaluation of perioperative dynamics and correlation analyses.

To assess the relationship between inflammatory and haemostatic parameters, a correlation analysis using Pearson’s coefficient (r) was performed for preoperative (admission) values only (Table 2). The analysis identified predominantly strong positive correlations (r = 0.65–0.93, *p* < 0.05 to *p* < 0.001) between inflammatory markers (CRP, ESR, haptoglobin, and seromucoids) and haemostatic indicators (fibrinogen, D-dimer, soluble fibrin complexes, and fibrinolytic activity). No pooling of postoperative data was performed. While most associations reached statistical significance, minor variations were observed in correlation strength across parameters, and not all relationships were uniformly significant.

Notably, a very strong positive correlation was found between inflammatory markers and indicators of hypercoagulability, supporting the hypothesis of an interdependent relationship between systemic inflammation and haemostatic disturbances in the context of severe trauma, including combat-related injuries to large joints.

In cases of significant tissue damage, including combat-related injuries to large joints, the central link connecting metabolic disturbances and alterations in fibrinolytic activity appears to be plasminogen—a plasma protein that functions as a key component in both primary and secondary fibrinolysis. Plasminogen also serves as a potent activator of metalloproteinases, which, when coupled with the initiation of inflammatory pathways, contribute to the degradation of collagen, elastin, and proteoglycans within the synovial joint system. This process can affect not only the injured joint but also distant sites throughout the body.

Upon activation by specific enzymatic factors, plasminogen is converted into plasmin, the active enzyme that initiates fibrinolysis and simultaneously exacerbates inflammatory changes within joint tissues. As inflammatory–dystrophic processes progress, there is an accumulation of acute-phase glycoproteins in the serum, proteolysis inhibitors that include inhibitors of fibrinolysis. These glycoproteins bind and inactivate plasmin, thereby inhibiting fibrinolysis. Consequently, fibrinogen accumulates in the plasma, which leads to an apparent paradox: despite inhibition of fibrinolysis, laboratory indices such as fibrinolytic activity and soluble fibrin complex (SFC) concentration may increase indirectly.

This interplay suggests the emergence of a vicious cycle, wherein heightened inflammatory activity under conditions of trauma promotes a hypercoagulable state. The stronger the inflammatory response, the more pronounced the haemostatic imbalance becomes.

Effective correction of haemostatic disorders in patients with combat-related injuries of large joints should therefore include targeted management of fibrinolytic dysfunction, especially in individuals with concurrent chronic liver or kidney diseases, which are associated with reduced circulating plasminogen levels. Monitoring anticoagulant therapy in such cases should rely on a combination of standard laboratory markers of inflammatory–dystrophic joint processes and comprehensive indicators of haemostasis, including parameters reflective of fibrinolytic and coagulatory status.

## 4. Discussion

The analysis of haemostatic biochemistry in patients with combat-related injuries to large joints revealed that even prior to the initiation of active therapeutic measures—including surgery—these individuals exhibited signs of a hypercoagulable state involving multiple components of the musculoskeletal and vascular systems. Previous studies by the authors have already reported that the coagulogram profile in such patients, even before surgical intervention, was indicative of thrombophilia [30].

Hypercoagulation is a nearly universal response in open traumatic injuries, particularly in the context of combat trauma. This state is part of an adaptive physiological response aimed at preventing blood loss and is primarily driven by extensive endothelial damage. In addition to vascular injury, the physiological stress response also plays a contributory role. As reported by Prete et al., cortisol levels rise sharply following orthopaedic surgeries such as total hip or knee arthroplasty and typically return to baseline within 24–48 h [31]. Similarly, Poredos et al. found elevated cortisol concentrations 24 h post-surgery, further supporting the involvement of the stress axis in modulating coagulation and inflammation [32].

In the present cohort, this stress-induced response was superimposed on an already activated haemostatic system, potentially intensifying the hypercoagulable state and heightening the risk of thromboembolic complications. Moreover, progressive consumption of coagulation factors may predispose to disseminated intravascular coagulation (DIC). Bawa et al. have noted that patients exhibiting preoperative hypercoagulability were more likely to develop postoperative deep vein thrombosis [33].

Furthermore, elevated levels of biochemical markers of inflammation were detected preoperatively in these patients. Surgical intervention, which represents a second wave of tissue injury, further exacerbated the inflammatory response. Given the bidirectional and interconnected nature of the inflammatory and endocrine systems, it is reasonable to expect activation of both pathways in the postoperative period [34].

The authors hypothesise that the thrombophilic state observed preoperatively in patients with combat joint trauma may have originated, at least in part, from systemic inflammatory activation. In turn, this inflammation may have amplified coagulation activity in a feedback loop, contributing to a “vicious cycle.”

The present findings support this mechanism: marked perioperative elevations of fibrinogen, D-dimer, soluble fibrin complexes, and C-reactive protein were accompanied by reduced antithrombin III activity, suggesting concurrent activation of coagulation and inhibition of natural anticoagulant pathways. The persistence and postoperative intensification of these abnormalities further confirm a reciprocal amplification between inflammatory and haemostatic responses. Such a mechanism aligns with the current understanding of thromboinflammation, whereby cytokine-mediated endothelial activation promotes fibrin deposition and impaired fibrinolysis, perpetuating vascular injury and hypercoagulability.

Elevated fibrinogen levels (hyperfibrinogenaemia), in particular, appeared to serve both as an acute-phase reactant and a procoagulant factor. This observation aligns with the findings of Burbul et al., who noted a pronounced rise in fibrinogen levels following major joint surgeries, identifying it as a key acute-phase protein [34].

Elevated leukocyte shift index (LSI) reflected the presence of active inflammation and diminished systemic reactivity in the acute phase. Simultaneously, an increase in the leukocyte index of intoxication (LII) pointed to rising endogenous intoxication and tissue breakdown, confirming the inflammatory burden at the systemic level [28]. The evaluation of peripheral blood leukocyte composition can, therefore, serve as a valuable tool in assessing systemic intoxication across various pathological conditions.

These findings support the rationale for implementing individualised and regulated haemostasis modulation strategies in patients with combat-related joint injuries. The goal would be to balance the natural need for clot formation to control bleeding while mitigating the risk of life-threatening thrombotic complications in the postoperative period. Such strategies should consider not only the volume of blood loss and extent of injury and surgical intervention, but more importantly, the biochemical indicators of haemostasis and inflammatory markers unique to each patient.

From a clinical standpoint, these findings highlight the potential utility of perioperative monitoring of fibrinogen, D-dimer, soluble fibrin complexes, and C-reactive protein as early indicators of thromboinflammatory risk. Patients showing markedly elevated levels—such as fibrinogen exceeding 6 g/L, D-dimer above 1.0 µg/mL, or CRP above 40 mg/L—may benefit from intensified thromboprophylaxis or closer postoperative surveillance for thromboembolic events. Given that postoperative day 1 represents an early and dynamic phase, serial measurements on admission, day 1, and day 3–5 could provide a more accurate assessment of persistent hypercoagulability and guide adjustments to anticoagulant therapy. Incorporating such laboratory-based risk stratification into perioperative management may help individualise prophylactic regimens and optimise outcomes in high-risk combat trauma patients.

The present study has several limitations. First, the sample size was relatively small (n = 29) and derived from a single specialised centre, which may limit the generalisability of the findings.

Second, the observation period was short, covering only admission and the first postoperative day, without longer follow-up to capture delayed thromboembolic or infectious complications such as venous thromboembolism (VTE), disseminated intravascular coagulation (DIC), or wound infection.

Third, perioperative factors—including anaesthetic technique, use of tranexamic acid, thromboprophylactic regimen, transfusion volume, and surgical extent—were not documented in detail and may have influenced biomarker dynamics.

Finally, the absence of hard clinical outcomes precludes conclusions regarding the predictive value of the observed biomarker changes.

Future multicentre studies with larger cohorts and extended postoperative monitoring are required to validate these preliminary findings and establish clinically actionable thresholds for risk stratification.

## 5. Conclusions

1. Upon admission, patients with combat-related injuries of large joints demonstrated significantly elevated levels of inflammatory markers alongside biochemical signs of activated coagulation and suppressed fibrinolysis. These findings reflect a state of thrombophilic predisposition in this patient population.
2. Surgical treatment in these patients was associated with a further increase in coagulation parameters and additional suppression of fibrinolytic activity, occurring in parallel with an intensified systemic inflammatory response.
3. Based on the biochemical analysis of haemostasis and inflammation markers, the most informative indicators for preoperative assessment of thrombophilic risk were identified as: elevated C-reactive protein (CRP, 37.75 ± 9.35 mg/L; +7.4-fold vs. controls), prolonged fibrinolysis duration (19.40 ± 0.60 min; +2.0-fold), increased fibrinogen (5.10 ± 0.47 g/L; +2.0-fold), D-dimer (426.15 ± 21.66 µg/L; +2.4-fold), and soluble fibrin complexes (23.22 ± 1.74 mg/L; +6.0-fold). These changes were accompanied by increased haptoglobin (1.85 ± 0.12 g/L; +2.6-fold) and erythrocyte sedimentation rate (38.9 ± 12.6 mm/h; +3.1-fold). Such levels indicate pronounced systemic inflammation and hypercoagulability that may predispose to perioperative thromboembolic complications, warranting enhanced laboratory surveillance and timely prophylactic intervention.
4. A high degree of correlation was observed between biochemical and clinical inflammatory markers and haemostasis parameters, both upon admission and on the first postoperative day, suggesting a tight interrelationship between inflammation and coagulation dysregulation in this cohort.
5. To adequately assess individual haemostatic status in patients with combat-related joint trauma, a combined approach using routine biochemical assays—including coagulogram and standard haematological tests—was found to be informative and practical in clinical settings.
6. In order to prevent postoperative hypercoagulable complications in patients with combat joint trauma, it is recommended to perform early monitoring of key haemostasis parameters and inflammatory markers prior to surgery. This should be accompanied by the implementation of tailored thromboprophylactic measures at pre-, intra-, and postoperative stages of care.

Future research should focus on translating these biochemical findings into clinically applicable tools for early risk stratification and perioperative decision-making. In particular, integration of dynamic biomarker monitoring with artificial intelligence (AI)-driven predictive models may enhance personalised thromboprophylactic management and improve outcomes in patients with combat-related musculoskeletal trauma.

## Figures and Tables

**Table 1:** Biochemical markers in patients with combat-related large joint injuries at admission and one day following surgical intervention.

Biochemical Parameter	Control Group (n = 30)	Patients at Admission (n = 29)	Patients One Day After Surgery (n = 29)
Prothrombin time (s)	14.40 ± 0.34	11.59 ± 0.83–19.51% ^(1)(4)^	11.02 ± 0.64–23.47% ^(1)(4)^ − 5.00% ^(2)(3)^
International normalised ratio (INR)	1.14 ± 0.07	0.97 ± 0.06–14.92% ^(1)(3)^	0.90 ± 0.06–21.05% ^(1)(4)^ − 7.22% ^(2)(3)^
Activated partial thromboplastin time (s)	29.50 ± 5.00	26.50 ± 1.10–10.17% ^(1)(3)^	23.05 ± 1.07–22.00% ^(1)(4)^ − 13.00% ^(2)(3)^
Fibrinogen concentration (g/L)	2.52 ± 0.12	5.10 ± 0.47 + 102.38% ^(1)(6)^	7.32 ± 0.50 + 190.48% ^(1)(6)^ + 44.00% ^(2)(5)^
Fibrinolytic activity (min)	6.53 ± 0.34	19.40 ± 0.60 + 197.09% ^(1)(6)^	23.45 ± 0.71 + 259.00% ^(1)(6)^ + 20.88% ^(2)(4)^
Soluble fibrin complexes (mg/L)	3.33 ± 0.05	23.22 ± 1.74 + 597.30% ^(1)(6)^	27.56 ± 1.89 + 727.63% ^(1)(6)^ + 18.69% ^(2)(4)^
D-dimer concentration (µg/L DDU)	180.14 ± 11.25	426.15 ± 21.66 + 136.57% ^(1)(6)^	531.28 ± 24.01 + 194.93% ^(1)(6)^ + 24.67% ^(2)(4)^
Antithrombin III activity (%)	95.4 ± 2.6	57.7 ± 2.6–39.52% ^(1)(5)^	50.1 ± 2.4–47.48% ^(1)(5)^ − 13.17% ^(2)(3)^
Sialic acid concentration (mmol/L)	1.91 ± 0.17	3.14 ± 0.19 + 64.40% ^(1)(6)^	3.46 ± 0.27 + 81.15% ^(1)(6)^ + 10.19% ^(2)(3)^
Seromucoids (g/L)	0.38 ± 0.06	0.66 ± 0.07 + 73.68% ^(1)(6)^	0.84 ± 0.08 + 121.05% ^(1)(6)^ + 27.27% ^(2)(4)^
Haptoglobin concentration (g/L)	0.70 ± 0.02	1.85 ± 0.12 + 164.29% ^(1)(6)^	2.14 ± 0.14 + 205.71% ^(1)(6)^ + 15.68% ^(2)(3)^
C-reactive protein (mg/L)	4.50 ± 0.62	37.75 ± 9.35 + 738.89% ^(1)(6)^	45.34 ± 7.35 + 908.00% ^(1)(6)^ + 20.11% ^(2)(4)^
Erythrocyte sedimentation rate (mm/h)	9.4 ± 3.1	38.9 ± 12.6 + 313.83% ^(1)(6)^	58.2 ± 14.5 + 519.15% ^(1)(6)^ + 49.61% ^(2)(5)^
Leukocyte shift index	2.419 ± 0.147	2.928 ± 0.165 + 21.04% ^(1)(4)^	3.564 ± 0.160 + 47.33% ^(1)(5)^ + 21.72% ^(2)(4)^
Leukocyte intoxication index	0.796 ± 0.108	1.997 ± 0.084 + 150.88% ^(1)(6)^	2.314 ± 0.117 + 190.70% ^(1)(6)^ + 15.87% ^(2)(3)^

^(1)^ Difference compared to the control group; ^(2)^ difference compared to values before surgical treatment; ^(3)^ not statistically significant (*p* > 0.05); ^(4)^ statistically significant (*p* < 0.05); ^(5)^ statistically significant (*p* < 0.01); ^(6)^ highly statistically significant (*p* < 0.001). Soluble fibrin complexes (SFCs) measured using Granum (Ukraine) reagents; results expressed in mg/L according to the manufacturer’s manual. Units follow standard laboratory conventions (s, min, g/L, mg/L, µg/mL, mmol/L, %).

**Table 2:** Correlations between inflammatory markers and haemostasis parameters in patients with large joint injuries prior to surgical treatment.

	Concentration of Sialic Acid	Concentration of Seroglycoid Acid	Concentration of Haptoglobin	Concentration of C-Reactive Protein	Erythrocyte Sedimentation Rate (ESR)	Leukocyte Shift Index (LSI)	Leukocyte Index of Intoxication (LII)
Prothrombin time (PT)	0.75	0.65	0.80	0.70	0.75	0.70	0.75
International normalised ratio (INR)	0.75	0.65	0.80	0.70	0.65	0.70	0.65
Activated partial thromboplastin time (aPTT)	0.650	0.65	0.68	0.70	0.70	0.65	0.70
Fibrinogen concentration (g/L)	0.88	0.80	0.85	0.88	0.93	0.90	0.90
Fibrinolytic activity (min)	0.60	0.62	0.65	0.65	0.65	0.67	0.65
Soluble fibrin complex concentration (mg/L)	0.85	0.75	0.85	0.85	0.75	0.70	0.75
D-dimer concentration (µg/L, D-dimer units)	0.75	0.80	0.80	0.85	0.90	0.90	0.90
Antithrombin III activity (%)	0.75	0.75	0.70	0.85	0.80	0.75	0.80

Correlation analysis performed for preoperative (admission) data only using Pearson’s coefficient (r). All correlations shown are statistically significant (*p* < 0.05–0.001). Soluble fibrin complexes (SFCs) measured in mg/L using Granum (Ukraine) reagents.

## Data Availability

The data supporting the findings of this study are available from the corresponding author upon reasonable request. The data are not publicly available due to privacy and ethical restrictions.

## Abbreviations

The following abbreviations are used in this manuscript:

AI	Artificial Intelligence
aPTT	Activated Partial Thromboplastin Time
AT-III	Antithrombin III
CRP	C-Reactive Protein
DD	D-Dimer
ESR	Erythrocyte Sedimentation Rate
FA	Fibrinolytic Activity
FG	Fibrinogen
HG	Haptoglobin
INR	International Normalised Ratio
LII	Leukocyte Index of Intoxication
LSI	Leukocyte Shift Index
PT	Prothrombin Time
SAs	Sialic Acids
SFCs	Soluble Fibrin Complexes
SGs	Seroglycoids
THA	Total Hip Arthroplasty
TKA	Total Knee Arthroplasty
TXA	Tranexamic Acid
